# Current Evidence on Cell Death in Preterm Brain Injury in Human and Preclinical Models

**DOI:** 10.3389/fcell.2020.00027

**Published:** 2020-02-18

**Authors:** Anita C. Truttmann, Vanessa Ginet, Julien Puyal

**Affiliations:** ^1^Clinic of Neonatology, Department of Women, Mother and Child, University Hospital Center of Vaud, Lausanne, Switzerland; ^2^Department of Fundamental Neurosciences, University of Lausanne, Lausanne, Switzerland; ^3^CURML, University Center of Legal Medicine, Lausanne University Hospital, Lausanne, Switzerland

**Keywords:** autophagy, apoptosis, necrosis, neonatal, neuroprotection, autophagic cell death, periventricular leucomalacia

## Abstract

Despite tremendous advances in neonatal intensive care over the past 20 years, prematurity carries a high burden of neurological morbidity lasting lifelong. The term encephalopathy of prematurity (EoP) coined by Volpe in 2009 encompasses all aspects of the now known effects of prematurity on the immature brain, including altered and disturbed development as well as specific lesional hallmarks. Understanding the way cells are damaged is crucial to design brain protective strategies, and in this purpose, preclinical models largely contribute to improve the comprehension of the cell death mechanisms. While neuronal cell death has been deeply investigated and characterized in (hypoxic–ischemic) encephalopathy of the newborn at term, little is known about the types of cell death occurring in preterm brain injury. Three main different morphological cell death types are observed in the immature brain, specifically in models of hypoxic–ischemic encephalopathy, namely, necrotic, apoptotic, and autophagic cell death. Features of all three types may be present in the same dying neuron. In preterm brain injury, description of cell death types is sparse, and cell loss primarily concerns immature oligodendrocytes and, infrequently, neurons. In the present review, we first shortly discuss the different main severe preterm brain injury conditions that have been reported to involve cell death, including periventricular leucomalacia (PVL), diffuse white matter injury (dWMI), and intraventricular hemorrhages, as well as potentially harmful iatrogenic conditions linked to premature birth (anesthesia and caffeine therapy). Then, we present an overview of current evidence concerning cell death in both clinical human tissue data and preclinical models by focusing on studies investigating the presence of cell death allowing discriminating between the types of cell death involved. We conclude that, to improve brain protective strategies, not only apoptosis but also other cell death (such as regulated necrotic and autophagic) pathways now need to be investigated together in order to consider all cell death mechanisms involved in the pathogenesis of preterm brain damage.

## Introduction

Neurodevelopmental deficits are frequent among infants born prematurely, particularly those born before 32 weeks of gestational age (very preterm infants, VPT). Around 5–10% VPT exhibit severe impairments such as cerebral palsy and cognitive delay, which are linked frequently to cystic periventricular leucomalacia (cPVL) and intraventricular and parenchymatous hemorrhages grades III and IV, corresponding to the most severe brain lesions. Around 10–15% of VPT exhibit more moderate impairments, such as limited cognitive functions and motoric deficits, linked morphologically to diffuse white and gray matter lesions. Finally, around 30–40% of VPT exhibit impaired academic achievement and behavioral disorders (Pierrat et al., 2017), associated presumably with altered brain development, dysmaturational disorders, and connectivity dysfunctions.

The major substrate of human preterm brain injury is the encephalopathy of prematurity (EoP) that is characterized by gray and white matter lesions reflecting combined acquired insults, altered developmental trajectories, and reparative phenomena (Kinney and Volpe, 2012). This term was quoted by Volpe in 2009 to characterize the multiple hit hypothesis and heterogeneous WM and GM impairments (including pre-oligodendrocytes injury, axonal injury, thalamic injury, subplate injury, and migrating GABAergic neuronal injury) affecting key developmental pathways and resulting in adverse clinical outcomes of VPT (Van’t Hooft et al., 2015; Schneider et al., 2016; Synnes and Hicks, 2018). On one hand, EoP covers severe brain lesions occurring after cell death such as in cPVL or intraventricular–parenchymatous hemorrhages (IVH). However, less severe brain lesions as in non-cystic and diffuse WM injury (detected by advanced MRI techniques and characterized by astrogliosis and microgliosis) are now more frequently observed with some features of cell death. On the other hand, subtler brain alterations can reflect disturbances of brain development or delay in brain maturation and affect functional connectivity with no apparent cell death (Ball et al., 2013; Gozdas et al., 2018).

The mostly studied anatomical structures involved in preterm brain injury are the periventricular white matter (PWM), the germinal matrix (source of most intraventricular hemorrhages), and the subventricular zone (SVZ, source of migrational and maturational disorders). Although there is growing evidence of the GM involvement (cortex, thalamus, nucleus caudatus, and deep GM injury), literature is sparser (Kinney and Volpe, 2012). Recent studies have shown that cortical interneurons development can be disrupted (reduced number and morphological complexity) in preterm infants with non-cystic WM injury or in inflammatory conditions (Panda et al., 2018; Stolp et al., 2019). Moreover, constant improvement in imaging techniques allows to study and detect microstructural alterations not only in WM but also in GM related to neurodevelopmental disorders (Chau et al., 2013; Nossin-Manor et al., 2013; Kersbergen et al., 2016).

Therefore, it is mandatory to understand the underlying pathophysiological cellular mechanisms contributing to WM and GM damage and/or organization defects. It is now widely recognized that events that induce an inflammatory context sensitize preterm brain to injury (Hagberg et al., 2015). Beside the inflammatory, vascular, and maturational factors implicated in the origin of preterm brain injury and some of those specific lesions, the etiology of the cellular factors and the cell death mechanisms encountered, either during altered brain development or in relation with specific lesions, are complex and interrelated. Focusing only on studies using cell death markers on pathological tissues, the present review proposes to do an overview of what has been described so far in human autopsy tissue and preclinical models regarding the different types of cell death involved in preterm brain injury. We here considered only studies using classical known and widely accepted cell death markers allowing the discrimination between cell death types (i.e., excluding studies reporting loss of cells without clarification of the type of cell death involved). Therefore, according to our selection criteria, this review will be focused mainly on the most severe cases of preterm brain injury involving “anatomical” evidence of brain damage.

Since EoP includes diverse causes that cannot be reproduced in one unique model, our investigations addressed the main pathological conditions related to EoP such as inflammation, hypoxia–ischemia (HI)/excitotoxicity, and other cytototoxic conditions related to current therapies such as hyperoxia, caffeine, and anesthetics. We will then discuss the need to investigate more in detail and improve our understanding of the different oligodendrocyte and neuronal death pathways that can play, even if not massive in some conditions, a role in neurological deficits developed by both preterm humans and animal models.

## Main Different Types of Cell Death

**FIGURE 1:**
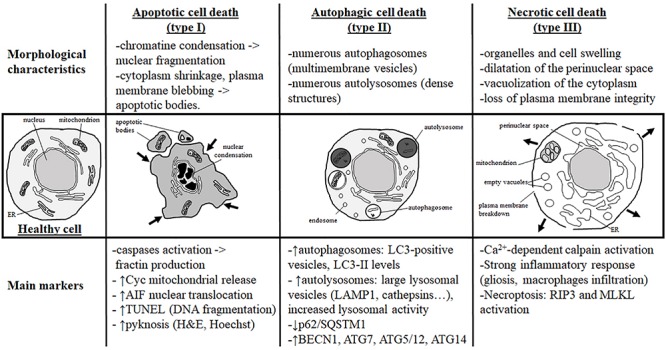
Morphological characteristics and most currently used markers of the three main types of cell death. Analysis of the dying cell ultrastructure is the most reliable method to identify the cell death type. The hallmark of apoptotic cell death (or type I) is a modification of the nuclear structure with presence of chromatin condensation and nuclear fragmentation. The cytoplasm shrinks and plasma membrane deforms until it forms apoptotic bodies that will be removed by phagocytic cells. Molecular markers of apoptosis are caspases (mainly caspase-3) and sometimes cytochrome c (Cyc) mitochondrial release in the cytosol. As a marker of caspase-independent apoptosis, nuclear translocation of AIF (Apoptosis-Inducing Factor) is mainly used. Pyknosis (condensation) of the nucleus shown by H&E (hematoxylin/eosin) or Hoechst stainings is also a main feature of apoptosis. TUNEL (Terminal deoxynucleotidyl transferase dUTP nick end labeling) is a staining revealing DNA fragmentation used as a marker of apoptosis with caution if not complemented with a cleaved-CASP3 immunostaining. Autophagic cell death (or type II) is mainly characterized by the presence of numerous autophagosomes (multimembrane vesicles containing intact cytoplasmic material such organelles) and autolysosomes (electron dense structure due to material at different stage of degradation). To conclude on autophagy flux with biochemical markers, it is necessary to study both autophagosome formation and autolysosome degradation. In autophagic cell death, both processes have to be enhanced. Autophagosome presence is reflected by the number of LC3-positive vesicles or by LC3-II level of expression on immunoblot. When autophagic degradation activity is enhanced, vesicles positive for lysosomal markers [such as LAMP1 (lysosomal-associated membrane protein 1) or cathepsins] are increased in number and size since autolysosomes are larger than primary lysosomes. The decrease in p62/SQSTM1 (Sequestosome-1), an autophagosome cargo protein degraded selectively by autophagy, reveals enhanced autophagy. In some cases, the level of autophagy-related (ATG) proteins such as BECN1 (BECLIN1/ATG6), ATG7, ATG5/ATG12, or ATG14 is increased. In necrotic cell death (or type III), ultrastructural changes are mainly cytoplasmic with organelles and cell swelling and presence of empty vacuoles. Perinuclear space is also dilated. Plasma membrane integrity can be finally lost, inducing a strong inflammatory response. Very few tools are available to identify necrotic cell death and mainly indirect markers are used such as strong inflammation response or Ca^2+^-dependent activation of calpains [mainly suggested by the production of a 145- to 150-kDa calpain-dependent cleavage of spectrin (fodrin)]. Necroptosis type of necrosis could be investigated by RIP3/RIPK3 (Receptor-interacting serine/threonine-protein kinase) or MLKL (mixed lineage kinase domain-like) expression and activation.

It is now globally accepted that three main morphological types of cell death exist as previously proposed by Peter Clarke in 1990 in physiological cell death conditions during development (Clarke, 1990): type 1 or apoptosis, type 2 or autophagic cell death, and type 3 or necrotic cell death (Figure 1). This classification is also valid for cell death involved in different pathologies, including brain injury. However, it has been described that, especially in the immature brain, hybrid morphological forms of cell death could occur in the same dying cell, reflecting the presence of interconnections between the molecular pathways controlling the execution of different cell death types (Portera-Cailliau et al., 1997; Puyal et al., 2013). The multiplicity of the cell death pathways and their positive/negative crosstalks that depend, moreover, on the cell type, their maturity, and the type of stress (intensity, duration, hypoxic, ischemic, inflammatory, etc.) represent then a major challenge for the design of neuroprotective strategies (Puyal et al., 2013). We will do here a very brief summary of the three main types of cell death (reviewed in Northington et al., 2011; Puyal et al., 2013).

### Apoptotic Cell Death

Since about 50 years, apoptosis is largely the best-known and characterized form of programmed cell death, which has been proposed to be the most important mechanism of delayed cell death occurring in different physiological and pathological conditions, including the developing brain. The most obvious morphological changes occurring during apoptosis concern the nucleus. Chromatin is compacted and the nucleus condensates, leading to the characteristic apoptotic feature of pyknosis and finally nuclear fragmentation. The cytoplasm is also shrunk and plasma membrane is distorted, forming blebbs that will finally detach and then form apoptotic bodies (Figure 1).

At the molecular level, apoptosis has been extensively characterized and can occur through two different but convergent pathways: the intrinsic and the extrinsic pathways (Galluzzi et al., 2018).

The intrinsic apoptotic pathway, also called the mitochondrial pathway of apoptosis, involves mitochondria, which represent a reservoir of apoptotic factors (stored in the intermembrane space of mitochondria). The balance of pro- and anti-apoptotic proteins from the BCL2 protein family controls mitochondrial membrane integrity. These proteins are responsive to intracellular stress coming from mitochondria, endoplasmic reticulum (ER), cytoskeleton, or nucleus. Pro-apoptotic proteins are directly (BAX and BAK) or indirectly (BAD, PUMA, BIM) involved in mitochondrial membrane permeabilization. As anti-apoptotic proteins, BCL2, BCL-xL, or MCL1 prevents BAX and BAK-mediated permeabilization of the outer mitochondrial membrane and then the release of apoptotic factors in the cytosol. Among these factors, some, such as cytochrome c, SMAC/Diablo, and Omi/HtrA2, activate caspase-dependent pathways and others, such as AIF and endoG, will trigger DNA fragmentation independently of caspases.

Caspases, which are cysteine-aspartic proteases, are the main common effectors of both intrinsic and extrinsic apoptotic pathways. Caspases activation is then widely considered as a gold standard marker of apoptosis in many studies including those on human tissues. Initiator caspases (CASP), CASP2, -8, -9, -10, and -12, activate the executor caspases (CASP3, -6, and -7) (Riedl and Shi, 2004), which have as substrates many essential proteins such as structural proteins, enzymes involved in DNA repair, or transcription factors resulting in the morphological changes induced by apoptosis (cell shrinkage, membrane blebbing, chromatin condensation, and DNA fragmentation).

Whereas CASP9 is involved in the intrinsic pathway of apoptosis, CASP8 and 10 are the initiators of the extrinsic pathway. CASP8 and -10 activations depend on the binding of extracellular death signals (such as TNFα, FasL, and TRAIL) on “death” receptors, resulting in the formation of a death-inducing signaling complex (DISC) used as a platform for CASP8 autoactivation. CASP8 can then directly activate the executor caspases (CASP3, -6, and -7). However, CASP8 can also interfere with the intrinsic pathway of apoptosis by mediating the cleavage of BID and then producing a truncated form of BID (tBID) able to induce BAX anchoring to the mitochondrial membrane and subsequent membrane permeabilization.

Finally, the executor caspases (CASP3, -6, and -7) could also be activated by CASP12 in some ER stress conditions (oxydative stress, unfolded protein response, etc.).

### Autophagic Cell Death

**FIGURE 2:**
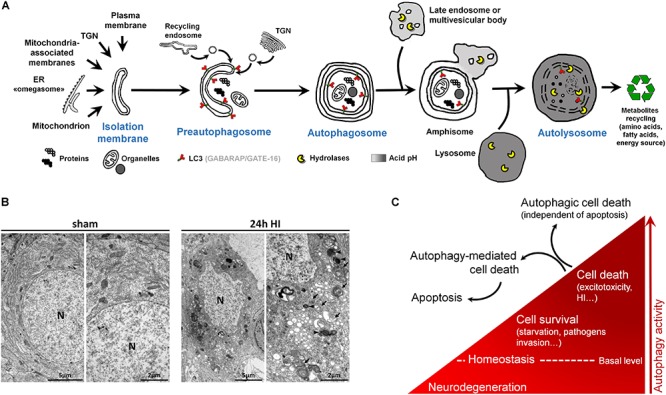
Autophagy and autophagic cell death. **(A)** Schematic illustration of (macro)autophagy degradation and recycling process showing the following: (1) isolation membrane formation from various possible intracellular membrane sources; (2) elongation and incurvation of the preautophagosome around the cytoplasmic material that has to be degraded (proteins, organelles); (3) closure end-to-end of the preautophagosome forming the autophagosome, a double-membrane vesicle containing undigested materials; (4) fusion of autophagosome with endosomes; (5) fusion of this amphisome with a lysosome to form a mature autophagosome able to degrade its content (autolysosome) thanks to lysosomal hydrolases. LC3 = Microtubule-associated protein 1A/1B-light chain 3 (and other family members). LC3 is the main marker of autophagosome. TGN = trans-Golgi network, ER = endoplasmic reticulum. **(B)** Electron micrographs showing CA3 neurons presenting features of autophagic cell death 24 h after perinatal hypoxia–ischemia (HI) in P7 rats (unilateral common carotid artery occlusion followed by 2 h of hypoxia at 8% of oxygen). Dying neurons displayed both numerous multimembrane vacuoles loaded with cytoplasmic material (autophagosomes, arrow) and electron dense vesicles containing digested material (autolysosomes, arrowhead). Note that nuclei (N) did not show chromatin condensation. Electron micrographs of CA3 neurons from sham-operated rat brain present healthy neurons. This study was carried out in accordance with the Swiss National Institutional Guidelines for Animal Experimentation. All experiments and methods were approved by the Veterinary Office of the Canton de Vaud. **(C)** Scheme illustrating the dual role of autophagy in neurons. At basal level, autophagy maintains cellular homeostasis. A reduction or impairment of autophagy can lead to neurodegeneration (such as for some proteinopathies). Autophagy can be activated, above its basal level, as a survival response to maintain energy level during a period of starvation, for example, or to eliminate invading bacteria or virus. However, in other stress conditions such as excitotoxicity, enhanced autophagy could lead to cell death as a mechanism of cell death by itself, independently of apoptosis or necrosis (autophagic cell death or type II), or as a mediator of another type of cell death, mainly apoptosis (autophagy-mediated cell death).

Autophagic cell death is morphologically characterized by the presence of numerous autophagosomes and autolysosomes in the cytoplasm of the dying cells, reflecting an excessive autophagy activity (Figures 1, 2).

Macroautophagy, the main type of autophagy (beside microautophagy and chaperone-mediated autophagy) (Cuervo, 2004), is a physiological intracellular catabolic process using lysosomes for degradation. Macroautophagy (hereafter called autophagy) consists in the engulfment of some cytoplasmic material that has to be degraded (such as impaired long-lived proteins and organelles), in an intermediate compartment termed autophagosome. Autophagy can be divided into three major steps: (1) the induction phase, which consists in the isolation of a membrane termed the preautophagosome; (2) the autophagosome formation, which involves elongation, incurvation, and finally closure of the preautophagosome, resulting in multimembrane vesicles containing cytoplasmic material including organelles; and (3) the autophagosome maturation, which consists in the fusion of the autophagosome with a lysosome leading to the formation of the functional degradative compartment of autophagy, the autolysosome (Figure 2A).

Besides, multiple analyses are needed to characterize the presence of autophagy (Klionsky et al., 2016), and the analysis of the autophagosomal marker, microtubule-associated protein 1 light chain 3 (LC3), is an essential step (Figure 2A). This ATG protein (homologue of yeast ATG8) is subject to an important ubiquitylation-like modification resulting in LC3 conjugation to a phosphatidylethanolamine (PE), a membrane phospholipid that allows LC3 to be incorporated into the membrane of early and late autophagosomes. Despite the fact that other methods could be used (such as the gold standard electron microscopy), an increased number of LC3-positive dots combined with an increased lysosomal activity (e.g., monitored by p62/SQSTM1 degradation) is, in many cases, sufficient to conclude for enhanced autophagy.

The recent discovery (since the 1990s) of more than 30 evolutionarily conserved autophagy-related (ATG) genes has largely contributed to understand the roles of autophagy in cell survival and death (Levine and Kroemer, 2019). Due to the important roles of autophagy in maintaining cell homeostasis and in cell adaptation to stress conditions (such as starvation or pathogen invasion) (Marino et al., 2011), the existence of a type of cell death mediated exclusively by autophagy has long been controversial (Debnath et al., 2005; Kroemer and Levine, 2008; Clarke and Puyal, 2012). However, it is now accepted that in specific conditions, autophagic cell death can occur, independently of necrosis and apoptosis, such as during development (morphogenesis or metamorphosis) or in particular stressful conditions (Clarke, 1990; Liu et al., 2013; Ginet et al., 2014b) (Figures 2B,C). Despite this, pure autophagic cell death is rarely observed, and there is now growing evidence that autophagy-mediated cell death is more frequently involved, meaning situations where autophagy is acting upstream of another type of cell death (Kriel and Loos, 2019), especially apoptosis (Grishchuk et al., 2011; Puyal et al., 2013) (Figure 2C).

### Necrotic Cell Death

Necrosis (or type 3B cell death) (Clarke, 1990) is usually considered as a passive form of cell death (unregulated and energy-independent) occuring rapidly and irreversibly in conditions precluding apoptotic and autophagic mechanisms (Golstein and Kroemer, 2007). Necrosis is characterized morphologically by the presence of dilated organelles (primarily mitochondria and ER), the vacuolization of the cytoplasm (empty vacuoles), and the swelling of the perinuclear space. The nucleus is first preserved; however, it ends up also swelling as the cell rounds up until plasma membrane ruptures (Figure 1), leading to the release of cellular contents [including damage-associated molecular patterns (DAMPs)] that promote and exacerbate the inflammatory response.

More recently, programmed forms of necrotic cell deaths have been identified, demonstrating that, in some cases, necrotic cell death could be regulated. One of the best characterized programmed form of necrosis is necroptosis. Necroptosis is initiated by the activation of death receptors (Kitanaka and Kuchino, 1999; Galluzzi et al., 2017) and is dependent on the RIPK1/3-MLKL pathway leading to the translocation of MLKL to intracellular and plasma membranes, and then inducing membrane permeabilization and rupture.

## Encephalopathy of the Prematurity in Humans and Evidence of Cell Death

As mentioned in the *Introduction*, EoP that covers the different injury patterns related to premature birth can affect either the WM or GM but, depending on the nature and the severity of the lesion, the extent of brain lesions could vary strongly between the individuals. With new emerging MRI techniques, better characterization of EoP now became possible and opens new understanding, such as functional connectivity, diffusion tensor imaging, fractional anisotropy measures and fiber tractography, and automated segmentation techniques for volume determination and growth (Ball et al., 2013; Gozdas et al., 2018; Schneider et al., 2018; Duerden et al., 2019; Jakab et al., 2019). The most prominent findings of EoP in VPT are (1) decreased global brain volume due to white (WM) and gray matter reduction [(GM); cortical GM as well as deep nuclear GM], (2) hydrocephalus ex vacuo (CSF), and (3) connectivity impairment (Dubois et al., 2008; Ball et al., 2013; Chau et al., 2013; Kidokoro et al., 2013; Friedrichs-Maeder et al., 2017; Gozdas et al., 2018; Duerden et al., 2019; Jakab et al., 2019).

This new understanding facilitates the development of new preclinical animal models, but still implementation of multiple features of EoP seems difficult (see review from Kinney and Volpe, 2012) and specific features of cell death in particular models, either neuronal or glial cell death, are lacking.

While it is widely accepted that cell death could happen in those conditions, cell death evidence in preterm brain injury are sparse and controversial, notably because few studies on human preterm brain tissue have characterized and determined either the type of cell death or, more importantly, the nature of the dying cell type (Tables 1, 2). Another point to mention is that in the studies claiming that cell death is not involved, not all the different types of cell death have been investigated (most often only apoptotic cell death markers were considered) and one can speculate that cell death types other than apoptosis might be involved in EoP. Finally, when comparing the different cohorts in Tables 1, 2, it is important to keep in mind that some infants have experienced a re-direction of care (most European countries) whereas some infants have died reaching their natural life span (US and Canada) and that the extent of cell death might have been influenced by different ethical practices. Moreover, as shown in Table 1, most of the studies were performed with cohorts from the Boston Children’s Hospital, meaning that some data could come from the same tissue collection.

**TABLE 1:** Cell death after WMI in human preterm brain from autopsy.

**Type of WMI**	**Country *(years of sample collection)***	**GA**	**PNA**	**Pathological features**	**Cell death markers**	**+ Cell types**	**ROIs**	**References**
Diffuse PWMI	US	Not indicated PCA: 28 to 42 w	Not indicated	↓ preOL (O4+), ↑ oxidative stress in O4+, no cortical neuronal loss	H&E, TUNEL c-CASP3, fractin IHC	O4	DWM	Back et al., 2005
Diffuse WMI	CA *(1983–2000)*	25 to 39 w	< 1 to 22 w	↓ immature OL, ↑ preOL (O4+ O1-); ↑ GFAP, CD44 and HA; ↑ Iba-1 necrotic foci (microcysts) 59% ↑ focal axonopathy	H&E c-CASP3 IHC axonopathy (β-APP, fractin)	n.d.	WM	Buser et al., 2012
Diffuse WMI	US *(2003-2010)*	22 to 42 w	< 1 to 9 w	↑ GFAP, CD44 and HA; ↑ Iba-1 necrotic foci (microcysts) 36% ↑ Olig2, ↑ focal axonopathy	H&E, c-CASP3 IHC, axonopathy (β-APP, fractin, SMI-312 IHC)	n.d.	WM	Buser et al., 2012
PVL Diffuse WMI	FR	-26 to 38 w -26 w	-0 d to 2.2 w -7 w	-cysts, ↑ Vimentin -↑ Vimentin	TUNEL	GFAP, Vimentin	CTX, PWM, SCWM	Gelot et al., 2009
PVL	US *(1997–1999)*	32.8 ± 3.1 w	3.7 ± 4.1 w	↑ WM and GM gliosis, focal necrosis; ↑ neuronal loss	H&E, GFAP IHC	n.d.	TH, Hipp, GP, cerebellar nuclei	Pierson et al., 2007
PVL	United Kingdom	24 to 28.9 w	1 d to 5.1 w	↑ GFAP, ↑ Iba-1, ↓ neurons, ↑ LC3-II	LC3-II IHC	n.d.	TH	Vontell et al., 2015
PVL	US	27 to 40 w	0 to 64 w	↑ gliosis (GFAP), ↑ CD68, ↑ protein nitration (O4+, O1+), ↑ oxidative stress (O4+, O1+ and GFAP+)	Hematoxylin, TUNEL, Oxydative stress	O4, O1	WM	Haynes et al., 2003
PVL	US	25 to 40 w	0 to 8 w	↑12/15-LOX (O4+, O1+ and APC+) in diffuse PVL component ↑12/15-LOX (CD68+) in cyst	H&E, TUNEL, Oxydative stress	n.d. and 12/15-LOX+	WM	Haynes and van Leyen, 2013
PVL	US	34.5 ± 1.1 w	7.0 ± 3.5 w	Diffuse astrogliosis no overall differences in olig2, MBP, c-CASP3 and KI67 ↑ c-CASP3 in necrotic foci	H&E c-CASP3 IHC	n.d.	PWM	Billiards et al., 2008
PVL	US*(1993–2007)*	32.5 ± 4.8 w	4.1 ± 6.6 w	↑ gliosis (GFAP), ↑ CD68, ↓ neuronal density, ↑ axonal damage	H&E, fractin IHC	n.d.	TH	Ligam et al., 2009
PVL	US	36 ± 3 w	7.5 ± 17 w	↑ gliosis (GFAP), ↑ axonopathy, ↑ GM damage (TH31%, CTX15%)	H&E, β-APP IHC, fractin IHC	n.d.	WM, TH, CTX	Haynes et al., 2008
PVL	US*(1993–2007)*	33.9 ± 4.3 w	5.9 ± 14.0 w	↓ layer V cortical neurons (MAP2+) No difference in cortical thickness ↑ cortical gliosis (GFAP)	H&E, fractin IHC	MAP2	CTX	Andiman et al., 2010
PVL	US	32.8 ± 4.1 w	1.9 ± 2.3 w	↓ granular neurons (MAP2+)	H&E	MAP2	CWM, PWM, SP	Kinney et al., 2012

*APC = adenomatous polyposis coli (mature OL); CA = Canada; CaR = calretinin; c-CASP = cleaved caspase; CTX = cerebral cortex; CWM = central white matter; d = day; DWM = deep white matter; FR = France; GA = gestational age at birth; GABA = g-aminobutyric acid; GAD = glutamic acid decarboxylase; GE = ganglionic eminence; GFAP = Glial fibrillary acidic protein; GM = gray matter; H&E = hematoxylin–eosin staining; Hipp = hippocampus; IHC = immunohistochemistry; LAMP1 = lysosomal-associated membrane protein 1; LC3 = microtubule-associated proteins 1A/1B light chain 3B; LOX = lipoxygenase; MAP2 = microtubule-associated protein 2; MBP = Myelin Basic Protein; n.d. = not defined; O1 = mature OL; O4 = immature preOL; OL = oligodendrocyte; PNA = postnatal age; PTL = perinatal telencephalic leukoencephalopathy; PVL = periventricular leukomalacia; PWM = periventricular white matter; PWMI = periventricular white matter injury; ROIs = regions of interest (i.e., where cell death is detected); SCWM = subcortical white matter; SP = subplate; TH = thalamus; United Kingdom = United Kingdom; US = United States; w = weeks; WM = white matter; WMI = white matter injury; + = positive.*

**TABLE 2:** Cell death after IVH in human preterm brains from autopsy.

**Type of insult**	**Country *(years of sample collection)***	**GA**	**PNA**	**Pathological features**	**Cell death markers**	**+ Cell types**	**ROIs**	**References**
IVH	DE + NL *(1999–2006)*	23 to 30 w	1 to 4 w	↑ sFas in CSF	sFas in CSF	–	CSF	Schmitz et al., 2011
	CA	18 to 28 w	1 to 98 d	↓ KI67+ cells, ↑ apoptosis	H&E, TUNEL, c-CASP3 IHC	n.d.	GE	Del Bigio, 2011
	US	23 to 27 w	1 to 5 d	↑ apoptosis, ↑ microglial infiltration (CD68+)	TUNEL, c-CASP3 IHC, Sytox green	n.d.	GM, PVZ	Georgiadis et al., 2008
	DE *(1999–2001)*	18 to 25 w	Not indicated	↑ sFas in CSF, no differences in sFasL and c-CASP3	sFas and sFasL c-CASP3 activity	–	CSF	Felderhoff-Mueser et al., 2003
	US *(1990–1998)*	26 ± 12 w	Severe IVH: 6 ± 6.5 d Mild IVH: 12 ± 5 d	↑ TUNEL (astrocytes and OL)	H&E, TUNEL	n.d.	WM	Chamnanvanakij et al., 2002
	HU *(1993–1999)*	24 to 36 w	3 d to 27 w	WM disorganization ↑ karyorrhexis	H&E, TUNEL	n.d.	WM, germinal layer	Hargitai et al., 2001

*CA = Canada; c-CASP = cleaved caspase; CSF cerebrospinal fluid; d = day; DE = Germany; sFas = soluble Fas; sFasL = soluble fasL; GA = gestational age at birth; GE = ganglionic eminence; GM = gray matter; H&E = hematoxylin–eosin staining; HU = Hungary; IHC = immunohistochemistry; IVH = intraventricular hemorrhage; n.d. = not defined; NL = Netherlands; PNA = postnatal age in weeks; PVZ = periventricular zone; ROIs = regions of interest (i.e., where cell death is detected); US = United States; w = weeks; WM = white matter; + = positive.*

### WM Injuries and Periventricular Leucomalacia (PVL)

The most important type of WM injury (WMI) in EoP is the PVL (malacia: infarction and leucos: white), first described as focal coagulative necrosis occurring in deep hemispheric WM (Banker and Larroche, 1962). The cPVL is the most severe form of PVL, whose incidence has decreased fivefold in the last decade and which carries the highest burden of neurological morbidity, especially cerebral palsy. Depending on the centers, the incidence of cPVL is around 4–8% for the babies born before 32 weeks of gestation and around 6 to 12% for babies born before 27 weeks of gestation (McGowan and Vohr, 2019).

cPVL is characterized by the presence of large focal periventricular WM necrotic lesions (necrotic foci), where all the cells are destroyed without specificity regarding the cell type (pan-cell death). These necrotic foci (evolving in cyst) are surrounded by reactive glial and microglial cells localized in the neighboring WM site where delayed cell death has been proposed to occur. Histological observations have reported that cPVL cases are characterized, in addition to the presence of necrotic foci, by a massive loss not only of WM (i.e., evidenced by the thinning of corpus callosum and subcortical WM) but also of GM (strong volume reduction of either cortical and deep nuclear structures). Several studies have reported increasing presence of apoptosis (mainly evidenced by TUNEL staining and c-CASP-3 immunohistochemistry) in cPVL in WM and GM, but few of these studies have identified the cell type of these apoptotic dying cells (Hargitai et al., 2001; Chamnanvanakij et al., 2002; Back et al., 2005; Robinson et al., 2006; Pierson et al., 2007; Billiards et al., 2008; Haynes et al., 2008; Ligam et al., 2009; Andiman et al., 2010; Zubiaurre-Elorza et al., 2011; Buser et al., 2012; Kinney et al., 2012; Haynes and van Leyen, 2013; Vontell et al., 2015) (see Table 1). However, apoptosis has been reported to mediate cell death of surrounding necrotic foci pre-oligodendrocytes (O4+ cells) (Haynes et al., 2003; Back et al., 2005; Robinson et al., 2006; Haynes and van Leyen, 2013), astrocytes (Gelot et al., 2009), but also of neurons in different locations including WM, subplate, cortex, basal ganglia, thalamus, and hippocampus (Robinson et al., 2006; Pierson et al., 2007; Ligam et al., 2009; Andiman et al., 2010; Kinney et al., 2012; Haynes and van Leyen, 2013; Vontell et al., 2015). Interestingly, the density of a type of subcortical neurons, granular neurons (GAD67/65+), is significantly reduced not only in the periventricular and central WM, but also in the subplate region in the presence of PVL, suggesting that this particular subtype of neurons is more selectively sensitive to preterm brain injury (Robinson et al., 2006). Then, diffuse axonal injury (distant from necrotic foci) is also observed in the WM and positive for the apoptotic marker fractin (Haynes et al., 2008; Andiman et al., 2010; Buser et al., 2012). This widespread axonopathy in PVL is reflecting either secondary degeneration to GM damage (such as thalamus) or primary alteration following a direct injury to the axon.

Focal or diffuse nondestructive lesions characterize a milder form of WMI with an incidence up to 20 to 25% in VPT infants (Schneider and Miller, 2019). This form is more frequently seen in preterm neonates in recent years and has been histologically characterized by the absence of focal necrotic lesions and the presence of diffuse reactive astrocytes (Billiards et al., 2008; Buser et al., 2012) and activated microglia (without macrophages infiltration) maintained in a pro-inflammatory state (Haynes et al., 2003; Buser et al., 2012). These observations are mostly confirmed by human imaging studies (Kidokoro et al., 2014; Van’t Hooft et al., 2015; Schneider et al., 2016; Synnes and Hicks, 2018), with microstructural alterations measured on advanced magnetic resonance (MR) techniques such as diffusion-weighted imaging (DWI) and apparent diffusion coefficients (ADC), T1 maps, and fractional anisotropy vectors (FA) and interesting longitudinal observations.

Few studies on postmortem tissue of human preterm suggested the absence of neuronal and cortical injury in diffuse WMI (dWMI) (Back et al., 2005; Robinson et al., 2006; Pierson et al., 2007) (see Table 1). Moreover, the study of Verney et al. (2012) reported that there was no significant loss of the number of Olig2+ oligodendrocytes in diffuse lesions (near necrotic foci) in preterm non-cystic PWMI (Verney et al., 2012). Their conclusion was that axonal injury could occur without pre-oligodendrocytes death. Billiards et al. (2008) showed also no decrease in Olig2+ cell density. However, they observed Olig2+ cell proliferation and an increase in Olig2+ cell density in necrotic foci and they assumed that if some loss of pre-oligodendrocytes still occurs, it could be compensated by a subsequent proliferation of OL progenitors (Billiards et al., 2008). Anyway, even if pre-oligodendrocytes death did not occur, this would not necessarily indicate that there is no cell death in PWMI (especially in foci). Billiards et al. (2008) showed the presence of apoptotic cells (without identifying the cell type) in necrotic foci (microcysts) and reported also that Olig2+ cell (that “did not appear apoptotic”) density was not affected in periventricular necrotic foci or in the surrounding WM. The study of the group of Charriaut-Marlangue showed that the majority of astrocytes “in the penumbral WM” were apoptotic (TUNEL+) in human preterm cPVL and in one case of non-cystic PVL (Gelot et al., 2009). Regarding these two studies, one can hypothesize that astrocytes could be the apoptotic cells described by Billiards and colleagues (2008). Another hypothesis could be that pre-oligodendrocytes death, even if not massive, is occurring through an apoptotic-independent mechanism, i.e., an alternative cell death pathway.

Apart from few studies reporting the loss of pre-oligodendrocytes (preOL) (Haynes et al., 2003; Haynes and van Leyen, 2013), it appears that the main mechanism of the glial dysfunction in dWMI is not due to glial cell death, but more due to dysmaturation of the oligodendrocyte precursor cells (PreO4^+^) and the imbalance and implication of “bad” microglia (M1) and “good” (M2) microglia phenotypes (Billiards et al., 2008; Buser et al., 2012). This disequilibrium seems to be responsible for the dysmyelination and the gliosis observed for instance in dWMI. Evidence of increased oxidative stress has been reported in immature OLs in dWMI, suggesting that oxidative stress could be a key player of the dysmaturation of OLs supporting the formation of dWMI lesions (Haynes et al., 2003; Billiards et al., 2008; Buser et al., 2012; Haynes and van Leyen, 2013). Multiple preclinical and clinical studies have shown that preOL are less resistant to radical stress, certainly because of decreased expression and function of antioxidant enzymes (such as SOD and GN-oxidase) compared to more mature babies, such as term or even later age (Van’t Hooft et al., 2015).

In summary, cell death is rarely reported in human preterm WMI and PVL tissue from autopsy and cell death characterization (co-localization of cell death and cell type markers) is either missing or sparse.

### Intraventricular and Intraparenchymatous Hemorrhages

Severe to moderate cerebral intraventricular hemorrhage (IVH grades III and IV or newly IPE) in preterm infants continues to be a major clinical problem and a neurological issue, occurring in about 5–10% of VPT. In contrast to cPVL, the incidence of IVH has remained stable over the last decade. Over 40% of surviving infants with IVH grade III develop post-hemorrhagic ventricular dilatation and about one third develop severe neurological impairment, such as cerebral palsy and mental retardation. To date, there is only little evidence for therapy to prevent infants from developing either hydrocephalus or serious neurological disability in this condition. There is one trial using a drainage and fibrinolytic approach (DRIFT Trial) that has shown some potential benefits on the neurological outcome, but data were inconclusive due to follow-up problems and other methodological issues (Luyt et al., 2019). There is also a management trial (ELVIS TRIAL) comparing early versus late approach in lumbar tapping and rickham insertion, which is underway (de Vries et al., 2019). It is known that blood rapidly accumulates within the ventricles following IVH, and this leads to disruption of normal cerebrospinal fluid flux and can cause obstruction and increased local tissue pressure as one of the potential etiological mechanisms (Klebe et al., 2020). As described in Table 2, reports about preterm human data, brain cell death, and IVH are rare, and consist in analyses of tissue from autopsy and clinical data in preterm infants, about apoptotic and necrotic markers detected in the cerebrospinal fluid (Felderhoff-Mueser et al., 2003; Schmitz et al., 2011), in the ganglionic eminence (Del Bigio, 2011), and in the periventricular zone (Hargitai et al., 2001; Chamnanvanakij et al., 2002; Georgiadis et al., 2008). However, to date, no study has systematically investigated the involvement of the different types of cell death and characterized the type of cell specifically affected by IVH.

### Iatrogenic Preterm Brain Injury

Iatrogenic conditions involved during prematurity are contributory factors changing the normal developmental trajectory of the brain, notably the impact of nutrition on growth and maturation (Beauport et al., 2017; Schneider et al., 2018) and the use of supplemental oxygen for instance. Further, some drugs such as caffeine as well as anesthetic and sedative drugs might influence physiological cerebral development. In regard to cell death types and iatrogenic factors, there are no direct reports in human tissue associating them with cell death types and cells involved, and only suppositions can be made based on preclinical studies as detailed extensively further.

#### Hyperoxia

When leaving the hypoxic intrauterine milieu, the preterm infant will be exposed to either diminished or excessive oxygen saturation and variation in cerebral blood flow, which are potentially harmful for the retina, the lung, but also the brain and the neurodevelopmental outcome at long term [Safe Boosc (Askie et al., 2011; Panfoli et al., 2018)]. Therefore, targeting specific oxygen saturation, and regional cerebral oxygenation levels through different methods such as near-infrared spectroscopy (NIRS) are widespread in the neonatal intensive care units, and several trials are still ongoing to confirm the detrimental effect of hypo/hyperoxia and free radical stress in preterm brain injury (Klebe et al., 2020). These data are supported by a tremendous work of body on preclinical models, which are described later.

#### Caffeine

Caffeine therapy for the apnea of prematurity syndrome (by stimulating the immature respiration center) is the most widespread medication used in premature infants. Babies start to be treated with caffeine between day of life 1 until they reach 34 weeks of corrected age and doses are relatively high (between 5 and 10 mg/kg/day 1 × daily). Clinical studies such as the CAP Trial (Caffeine for Apnea of Prematurity) have shown a protective effect of caffeine administrated in VPT infants, in terms of neurodevelopmental outcome and mortality (Schmidt et al., 2012, 2017; Panfoli et al., 2018). While the short-term data at 18 months was slightly convincing and significant, the effect about the neurodevelopmental outcome was ephemeral at 5 years of follow-up. In fact, a pilot study using high-dose caffeine therapy in premature infants showed increased cerebellar injury and neurobehavioral deficits when compared to low dose (McPherson et al., 2015). This controversy between preclinical worrying and clinical reassuring data is not really rational and raises many questions and doubts. There is probably an effect of the dose. Therapeutical levels preventing apnea consequences in babies are low and harmless, whereas higher doses and/or the multiplicity of drugs combined are linked to neurotoxicity, but this has still to be confirmed.

#### Anesthetics

When looking at clinical risk factors for neurodevelopmental outcome, surgery is a major and constant contributor for impaired neurological outcome, and these concerns are true for neonates at term and premature infants (Sun, 2010; Morriss et al., 2014; Sinner et al., 2014; Stolwijk et al., 2016; Walker et al., 2018). Premature infants may undergo several surgical procedures such as ductus ligation, laparotomies, and thoracotomies defined as major surgeries and herniotomies or endoscopic laryngoscopies defined as minor surgeries. It is still unclear whether the surgical stress, the pain perception, or the administrated drugs for anesthesia are neurotoxic (Broad et al., 2017; Walker et al., 2018), but the combination of both seems to play a major role. This also explains the development of several preclinical models in rodent and primate animals in order to answer the unsolved questions (Baud and Saint-Faust, 2019).

There are only very few clinical data on humans and associated secondary effects with anesthetics/or surgery. The group of Buonocuore measured oxidative stress (Stolwijk et al., 2017), using non-protein bound iron (NPBI) in neonates who underwent noncardiac surgery. These biomarkers were found to be predictive for brain injury post-operatively. Walker et al. (2018) in a cohort of United Kingdom Epicure in ELBW infants found an association of neonatal surgery in the volume of the amygdala and the somatosensory response and pain response in young adults ex-preterm. Filan et al. (2012) analyzed *post hoc* 30 babies exposed to surgery who had more evidence of WMI and smaller brain volumes, particularly in the deep GM. They could not find any difference in the neurodevelopmental outcome.

The most used anesthetic drugs in premature infants are GABA agonists such as thiopental and propofol, and NMDA antagonists (either ketamine or anesthetic gazes such as isoflurane or sevoflurane). Potential effects of anesthetic drugs on the brain development are driven from the multiplicity of preclinical studies, but very few are described on human data (Sun, 2010; Sinner et al., 2014).

## Preclinical Models of Preterm Brain Injury and Evidence of Cell Death

Due to the multifactorial etiologies and the heterogeneity of the brain pathologies involved in EoP, the challenge was, over the last decades, to develop preclinical models reproducing as best as possible the pathophysiology of brain injury and/or brain development impairment observed in the premature human brain. However, the use of a single preclinical model of preterm brain injury cannot reproduce exactly the human situation, and in conclusion, each model presents advantages/disadvantages as reviewed by others (Back et al., 2012; Kinney and Volpe, 2012; Jantzie and Robinson, 2015).

Cell death has been investigated in preterm models in three main species: rodent (rat and mice), sheep, and non-human primate (macaque, baboon) (Tables 3–9). Few studies were undertaken in rabbit and pig. Human preterm neonates are highly exposed to WM injury between 23 and 32 weeks of gestation that corresponds in rodents to the postnatal period before 7 days (P1–P6), in sheep to gestational age around 95 days (90–120 days, term at 145 days), and in macaques to gestational age 125–145 days (term at around 160 days). Whereas substantial cell death can occur in severe models such as those of cPVL in rodents (Table 3), the identification of the presence of cell death frequently showed sparse cell death in specific sites both in WM and GM affecting mainly oligodendrocytes (OL) but also neurons. Animal models have contributed to hypothesize that, in preterm brain injury, a primary OL loss during the “acute” phase is followed by a regenerative process producing immature OL that leads to impaired myelination (Rousset et al., 2006; Segovia et al., 2008). Neuronal injury, including not only neuronal death but also deficits in dendrite and spine maturation (Balakrishnan et al., 2013), could be a direct consequence of primary GM injury as well as secondary through axonal deleterious factors linked to primary WM injury (Rousset et al., 2006; Segovia et al., 2008; Back and Volpe, 2018).

**TABLE 3:** Cell death in preclinical models of preterm white matter injury following inflammation.

**Species**	**Time of the insult**	**Model**	**Cell death markers (time post insult)**	**+ Cell types**	**ROIs**	**Neuroprotectant**	**References**
Sheep	91 d ga	LPS (150 ng/kg), i.v., over 3 d	c-CASP3 (7 d)	n.d.	SCWM	hUCB cells	Paton et al., 2018
	93–96 d ga	LPS (100 ng), i.v.	TUNEL, c-CASP3 (3 d), Cysts (30%)	Olig2, CNPase	CBWM	–	Dean et al., 2009
	109 d ga	LPS (150 ng/kg), i.v., over 3 d	Pyknotic nuclei (114 d ga)	n.d.	PVWM	hAECs	Yawno et al., 2017b
	5 h, 12 h, 24 h, 2 d, 4 d, 8 d, or 15 d before preterm delivery at 125 d ga	1 × LPS (10 mg), intra-amniotic	c-CASP3 (2 d to 15 d)	n.d.	WM, Hipp, CX	–	Gussenhoven et al., 2018

Rat	E17, 6 h before preterm delivery	LPS (1 mg/kg), maternal i.p.	c-CASP3 (6 h)	n.d.	n.d.	Fingolimod	Yavuz et al., 2017
	E18	LPS (1 mg/kg), maternal i.p.	TUNEL (E20)	PDGFαR	SZ	NAC	Paintlia et al., 2004
	E18+E19	LPS (0.5 mg/kg.), maternal i.p., LPS (0.3 mg/kg.), maternal i.p.	-TUNEL, c-CASP3 (P6) -TUNEL, c-CASP3 (P7) -Impaired autophagy (P1)	n.d.	-PVWM -ST, PVWM, GV -Whole brain	-APC, EPO, SPD – –	Kumral et al., 2007, 2017, [Bibr B221][Bibr B172] Carloni et al., 2016
	P3	LPS, i.c. -(10 μg) -(1 mg/kg)	-Fractin (4 d) -RIP1, RIP3 and MLKL RNAs (1 d) -Fractin (1 d)	-SMI-312	-ST, -ST, SCWM, CC -Whole hemisphere -CG, CC	–	Ginet et al., 2016, [Bibr B136][Bibr B161]
	P3	LPS (0.25 mg/kg), i.p.	-c-CASP3, TUNEL (2d)	n.d.	-CX, SCWM extracts	-MSC-EVs	Drommelschmidt et al., 2017
	P5	LPS (10 μg), i.c.	-TUNEL (1 d) -Cysts (30%, injection site)	n.d.	-SCWM (near injection) -CC	-Minocycline –	Fan et al., 2005a
	P5	LPS (2 mg/kg), i.p.	TUNEL, c-CASP3 (1 d)	O4	CG	Celecoxib	Fan et al., 2013
	P5	IL-1β (10 ng), i.c.	-TUNEL, c-CASP3 (1d)	Lectin, O4, O1, NF	-Sub-ventricular area, CC, outer layers of CTX	-NBQX	Cai et al., 2004

Rabbit	24–30 d ga	LPS, uterine horn, + ceftriaxone (+24 h)	-TUNEL (2 d) -Cysts (∼25–23%)	n.d.	-PVWM, CA2 -PVWM	–	Debillon et al., 2003, Debillon et al., 2000

Rhesus macaque	130 d ga, 16 h before preterm delivery	LPS (1 mg), i.a.	c-CASP3	n.d.	PVWM	–	Schmidt et al., 2016

*APC = activated protein C; CA = cornu ammonis; CBWM = cerebellar WM; c-CASP3 = cleaved caspase-3; CNPase = 2′,3′-cyclic nucleotide 3′-phosphodiesterase; CTX = cortex; d = day; E = embryonic day; ga = gestational age; GV = germinative ventricular zone; hAECs = human amnion epithelial cells; h = hours; Hipp = hippocampus; i.a. = intra-amniotic; i.c. = intracerebral; i.p. = intraperitoneal; i.v. = intravenously; LPS = lipopolysaccharide; MSC-EVs = mesenchymal stem/stromal cells; NAC = N-acetyl cysteine; n.d. = not defined; NF = neurofilament; O4, O1 = oligodendrocyte markers O4, O1; Olig2 = oligodendrocyte transcription factor; P = postnatal day; PDGFαR = platelet-derived growth factor-α receptor; PVWM = periventricular WM; ROIs = regions of interest (i.e., where cell death is detected); SCWM = subcortical white matter; SMI-312 = anti-neurofilament Marker (pan axonal, cocktail); SPD = surfactant protein; ST = striatum; SZ = subventricular zone; TH = thalamus; hUCB = human umbilical cord blood; WM = white matter; nd = not defined.*

**TABLE 4:** Cell death in preclinical models of preterm brain injury following hypoxia/ischemia.

**Species**	**Time of the insult**	**Model**	**Cell death markers (time post insult)**	**+ Cell types**	**ROIs**	**Neuroprotectant**	**References**
Sheep	91 d ga	biCCAO (45 min)	-TUNEL (1 d) -c-CASP3, fractin, pyknotic nuclei	-O4,O1 -n.d.	-PVWM, CTX, BG -PVWM	–	Riddle et al., 2006
	92 d ga	- Maternal Hx (10.5% O_2_, 30 min) + brachioc. AO (25 min) - + 2^nd^ Hx (+6 d)	-c-CASP3 (1 d) pyknotic nuclei (1 d, 4 w) -“necrotic” foci (H&E, Iba1+)	- O4 - n.d.	-PVWM, SP -PVWM	–	McClendon et al., 2017 Hagen et al., 2014
	94–96 d ga 93–96 d ga	UCO (25 min)	-TUNEL (3 d) -cysts (∼30%)	-GFAP -n.d.	-SCWM, PVWM, hipp, STcn - PVWM	-Dopa –	Brew et al., 2016 Mallard et al., 2003
	98-99 d ga	UCO (25 min)	-c-CASP3 (3 d) -NeuN count (3 d) -c-CASP3, NeuN count (3 d)	n.d.	-STcn, STp, CA4, DG, MN, MGN, SCWM -STcn, CA1/2, CA3, CA4, DG, MN -CA3	-Epo -Hypoth. –	Wassink et al., 2017 Bennet et al., 2007 Dean et al., 2006
	102 d ga	UCO (23-25 min)	-c-CASP3, TUNEL (10 d) -NeuN count (10 d) -TUNEL (10 d)	n.d.	-SVZ, PVWM, SCWM, ST -CTX -iC	-Melatonin – -UCB	Yawno et al., 2017a Li J. et al., 2017 Li et al., 2016b
	125–133 d ga	Partial UCO (blood flow < 50%, 60 min)	-TUNEL (+3 h) -Necrotic-like neurons (H&E, Nissl) (0 h, 3 h), -TUNEL (3 h) -Annexin V and PI (0 h, 3 h)	n.d.	-CTX, Hipp, BG, CB, BS -CB, pons, BG, mesencephalon -CTX, CB, Pons -CTX, CB, HTH, TH, Hipp	-WIN 55, 212-2 –	Alonso-Alconada et al., 2010 Goni-de-Cerio et al., 2007
	132 d ga	UCO (10 min)	-“Necrotic”/ pyknotic cells (H&E) (1 d), -c-CASP3 (1 d)	n.d.	CC, PVWM, eC, CTX, SVZ	–	Castillo-Melendez et al., 2013

Rat	E5–E19	Maternal Hx (10% O_2_)	-Cysts (H&E), TUNEL (P0, P5)	n.d.	SCWM	–	Fontaine et al., 2008
	E18	- Uterine AsO (60 min) (45 min)	-TUNEL (P2) -c-CASP3 (P5, P9) -c-CASP3 (P2, P9)	-NeuN -n.d. -NeuN, O4,O1,PDGFαR	-SPN -PVWM -CTX, PVWM	-EPO -EPO –	Jantzie et al., 2015a Mazur et al., 2010 Robinson et al., 2005
	E21	Uterine horn blood supply Occ (10 or 15 min)	TUNEL, c-CASP3 (1 d)	n.d.	-Hipp, CTX	–	Huang et al., 2013
	P1	100% N_2_ (25 min)	-TUNEL (1 d) -FJB (1 d) -Necrosis,↑ autophagy	n.d.	-CA1, DG -CA2, CA3 -hipp	– – –	Takada et al., 2015; Ikebara et al., 2017
	P1–P2	UniCCAO+6%O_2_ - (3 h) - (1.5 h–2 h)	-ISEL staining (12 h, 1 d) -c-CASP3 (1 d)	n.d.	- SP, IZ, SVZ, neoCTX, TH -CTX	– –	McQuillen et al., 2003 Okusa et al., 2014
	P3	UniCCAO+6%O_2_ - (30 min) - (3.5 h)	-c-CASP3, fractin (1 d) -c-CASP3 (4 d)	-n.d. -O4	-CTX -SCWM	-Lf –	van de Looij et al., 2014 Segovia et al., 2008
	P6	uniCCAO+ 6%O_2_ (1 h)	TUNEL (1 d)	n.d.	periCCWM	MgSO_4_ (-30 min)	Seyama et al., 2018
Mouse	P3–P11	10.5% O_2_, for 8 d	-c-CASP3, PI (P11, P15, P18)	Olig, CC1	-CC, CG, eC	–	Jablonska et al., 2012; Scafidi et al., 2014
	P2–P3	UniCCAO+6%O_2_ (30 min)	TUNEL (1 d)	n.d.	CTX	Hypoth.	Kida et al., 2013

Pig	35/60 d ga	7% O_2_ (1 h)	BAD, BAX	n.d.	CTX	–	Abedin et al., 2005

Rabbit	E22/E25	Uterine ischemia (40 min)	-TUNEL (1 d) -c-CASP3 (1 d)	-n.d. -NeuN, Ctip2	-CTX, BG, TH, CXP, STp, SCWM -CTX, Hipp, ST, TH	– –	Derrick et al., 2004; Buser et al., 2010

*AO = artery occlusion; biCCAO = bilateral carotid artery occlusion; brachioc = brachiocephalic; BS = brainstem; BG = basal ganglia; CB = cerebellum; CBWM = cerebellar WM; CC = corpus callosum; c-CASP3 = cleaved caspase-3; CG = cingulum; CTX = cortex; CxP = cortical plate; d = day; DG = dentate gyrus; Dopa = dopamine; E = embryonic day; eC = external capsule; EPO = erythropoietin; ga = gestational age; GV = germinative ventricular zone; h = hours; H&E = hematoxylin–eosin staining, Hipp = hippocampus; HTH = hypothalamus; Hypoth. = hypothermia; Hx = hypoxia; iC = internal capsule; i.c. = intracerebral; i.p. = intraperitoneal; i.v. = intravenous; IZ = intermediate zone; Lf = lactoferrin; LPS = lipopolysaccharide; MGN = medial geniculate nucleus; MN = thalamic medial nucleus; n.d. = not defined; O = occlusion; P = postnatal day; periCCWM = pericallosal white matter; PI = propidium iodide; PVWM = periventricular WM; ROIs = regions of interest (i.e., where cell death is detected); SCWM = subcortical white matter; SP = subplate; SPN = subplate neurons; ST = striatum; STcn = striatal caudate nucleus; STp = striatal putamen; SZ = subventricular zone; TH = thalamus; UCO = umbilical cord occlusion; uniCCAO = unilateral Common Carotid Artery Occlusion; w = weeks.*

**TABLE 5:** Cell death in preclinical models of preterm LPS-sensitized HI brain injury.

**Species**	**Time of the insult**	**Model**	**Cell death markers (time post insult)**	**+ Cell types**	**ROIs**	**Neuroprotectant**	**References**
Mouse	P2	LPS (0.05 μg/kg, -3 h), i.p. + uniCCAO + 6.5% O_2_ (90 min)	c-CASP3 (1 d)	O4, RECA	SCWM	JNK inhibition	Wang et al., 2010, 2012
	P3	LPS (0.05 μg/kg, -2 h), i.p + uniCCAO + 8% O_2_ (20 min)	TUNEL, c-CASP3 (1 d)	n.d.	CTX	PINK1 KO	Zhu et al., 2016

*c-CASP3 = cleaved caspase-3; CTX = cortex; d = day; h = hours; i.p. = intraperitoneal; LPS = lipopolysaccharide; P = postnatal day; ROIs = regions of interest (i.e., where cell death is detected); SCWM = subcortical white matter; uniCCAO = unilateral Common Carotid Artery Occlusion; w = weeks.*

**TABLE 6:** Cell death in preclinical models of excitotoxin-induced preterm brain injury.

**Species**	**Time of the insult**	**Model**	**Cell death markers (time post insult)**	**+ Cell types**	**ROIs**	**Neuroprotectant**	**References**
Rat	P5	SCWM ibotenate injection (10 μg)	-c-CASP3, enhanced autophagy (1 d) -c-CASP3, Fluoro-Jade B, AIF (1 d), -cysts -c-CASP3, TUNEL (1 d)	-NeuN -n.d. -n.d. -n.d.	-CTX -CTX -PVWM -CTX	-3-MA -HIP/HAP; S18986, -BDNF -BDNF	Descloux et al., 2018 Destot-Wong et al., 2009; Haldipur et al., 2014 Husson et al., 2005

Mouse	P5	SCWM ibotenate injection (10 μg)	-c-CASP3, AIF (5 d) -c-CASP3, AIF, Fluoro-Jade B (1 d) -c-CASP3 (1 d, 2 d), cysts -c-CASP3 (1 d–30 d) -TUNEL -c-CASP3, TUNEL, Fluoro-Jade B (1 d) -TUNEL (1 d)	-n.d. -n.d. -n.d. -n.d. -GFAP -n.d. -n.d.	-CTX, SCWM -CTX -CTX -CTX -SCWM -CTX -CTX	- -FBP -CASP2 KO -Substance P - -NAP -melatonin	Griesmaier et al., 2010; Neubauer et al., 2016 Dommergues et al., 2003; Rogido et al., 2003; Wegleiter et al., 2014 Carlsson et al., 2011 Medja et al., 2006 Tahraoui et al., 2001 Sokolowska et al., 2011 Husson et al., 2002

*AIF = apoptosis-inducing factor; c-CASP3 = cleaved caspase-3; CTX = cortex; d = day; FBP = fructose 1,6-biphosphate; h = hours; HIP/PAP = hepatocarcinoma intestine pancreas protein/pancreatitis-associated protein I; NAP = actif motif of ADNP (activity-dependent neuroprotective protein); 3MA = 3-methyladenine; P = postnatal day; PVWM = periventricular WM; ROIs = regions of interest (i.e., where cell death is detected); SCWM = subcortical white matter.*

**TABLE 7:** Cell death in preclinical models of preterm brain injury following intracerebral hemorrhage.

**Species**	**Time of the insult**	**Model**	**Cell death markers (time post insult)**	**+ Cell types**	**ROIs**	**Neuroprotectant**	**References**
Mouse	P1	i.c. autologous blood (15 μl)	-TUNEL (2 d)	-n.d.	-ST, germinal matrix	-	Xue et al., 2003
	P5	i.c.v. autologous blood (20 μl)	-TUNEL (P23)	-n.d.	-PV SCWM	-UC-MSCs	Mukai et al., 2017

Rat	P4	dam’s blood (100 μl) i.c.v.	-TUNEL (P32)	-n.d.	-PVZ	-Human UC-MSCs	Ahn et al., 2013

Rabbit	29 d ga, (2 h, 3 h, or 4 h after preterm delivery	Glycerol (6.5 g/kg), i.p	-TUNEL -TUNEL -c-CASP3 (1 d) -TUNEL, Fluoro-Jade B, CASP 3/7 activity (3 d) -TUNEL, Fluoro-Jade B (1-3 d)	-O4 -O4 -n.d. -n.d. -n.d.	-CR, CC -CR, CC -Choroid plexus -PVA, CTX -PVA, CTX	-NBQX -NOG – -Celecoxib, SC-51089, etanercept -Apocynin	Dohare et al., 2016 Dummula et al., 2011 Gram et al., 2014 Vinukonda et al., 2010 Georgiadis et al., 2008; Zia et al., 2009

*BG = basal ganglia; CC = corpus callosum; CASP3 = cleaved caspase-3; CG = cingulum; CTX = cortex; CR = corona radiate; d = day; ga = gestational age; h = hours; i.c. = intracerebral; i.c.v. = intracerebroventricular; NOG = Noggin; P = postnatal day; PI = propidium iodide; PV = periventricular; PVA = periventricular area including germinal matrix, caudate nucleus, corona radiata, and corpus callosum; ROIs = regions of interest (i.e., where cell death is detected); SCWM = subcortical white matter; ST = striatum; UC-MSCs = umbilical cord-derived mesenchymal stromal cells.*

**TABLE 8:** Cell death in preclinical models of iatrogenic preterm brain injury: hyperoxia.

**Species**	**Time of the insult**	**Model**	**Cell death markers (time post insult)**	**+ Cell types**	**ROIs**	**Neuroprotectant**	**References**
Rat	E21	80% O_2_ (8 d)	-TUNEL (P3)	-n.d.	-WM	-iNO	Pham et al., 2014
	E21	60% O_2_ (8 d)	-TUNEL (P3)	-n.d.	-WM	-	Vottier et al., 2011
	P0	80% O_2_ (5 d)	-TUNEL, cell count (5 d)	-Neurons -Neurons	-CA1, CA3 -CA1, DG, prefrontal/ parietal/ retrosplenial CTX, subiculum	-Uridine -EPO -Topiramate	Goren et al., 2017 Yis et al., 2008a Yis et al., 2008b Kurul et al., 2009
	P2	95% O_2_ (5 d)	-ELISA DNA fragm.	-n.d.	-CB, basal FB, frontal CTX, Hipp	-BSO	Taglialatela et al., 1998
		80% O_2_ -(1 d)	-TUNEL, AIF (5 d) -c-CASP3 (1 d) -TUNEL -c-CASP3 -CASP2, 3, 8; silver staining -TUNEL (P7)	-CC1 -n.d. -n.d. -MBP -n.d. -Olig2	-CC, CG, eC -CTX, WM -frontal/ retrosplenial CTX, HTH, TH -CC -CTX, STcn, NAcc, CC and adjacent WM, TH, Hipp, HTH - CC, deep cortical WM, CTX, TH	-Minocycline -Dextromethorphan -Dexmedetomidine -*17*β-estradiol -EPO -EPO	Schmitz et al., 2014 Posod et al., 2014 Sifringer et al., 2015 Gerstner et al., 2006 Gerstner et al., 2007 Kaindl et al., 2008 Hoeber et al., 2016
	P6	-(1 d or 2 d)	-TUNEL, neuronal density -PARP-1, AIF, c-CASP3	-Nestin, DCX, NeuN -n.d.	-Frontal/ retrosplenial/parietal CTX, TH, HTH -Whole brain	-Caffeine -Caffeine	Endesfelder et al., 2014 Endesfelder et al., 2017b
		-(12 h or 1 d)	-CASP2, Cyc, APAF-1, AIF, BCL2, BID, c-CASP3, Fluoro-Jade B -BECN1, ATG3, ATG5, ATG12, LC3	-Neurons and n.d. -n.d.	-TH -Whole brain	-Caspase inhibitor (TRP601) -EPO	Sifringer et al., 2012 Bendix et al., 2012
		-(2 h, 6 h, 12 h, 1 d, 2 d, and 3 d)	-c-CASP8, c-CASP3, Fas, FasL, FADD -Fluoro-Jade B, silver staining, CASP1	-n.d. -n.d.	-CG, parietal CTX, CC, ST, TH	-CASP8 inhibitor (TRP801) -Recombinant IL-18BP	Dzietko et al., 2008 Felderhoff-Mueser et al., 2005

Mouse	P6	80% O_2_ -(2 h, 6 h, 12 h, 1 d, 2 d) -(1 d)	-Fluoro-Jade B, silver staining -CASP2, CASP3, CASP8, silver staining	-n.d. -n.d.	-CG, parietal CTX, CC, ST, TH -CTX, STcn, NAcc, CC and adjacent WM, TH, Hipp, HTH	-KO IRAK-4 -EPO	Felderhoff-Mueser et al., 2005 Kaindl et al., 2008
	P6	FasL + 80% O_2_ (1 d)	-c-CASP8, c-CASP3	-n.d.	-Cingulate/parietal CTX, CC, ST, TH	-CASP8 inhibitor (TRP801)	Dzietko et al., 2008

*AIF = apoptosis-inducing factor; BSO = buthionine sulfoxide; CC = corpus callosum; c-CASP = cleaved caspase; CG = cingulum; CTX = cortex; Cyc = cytochrome c; d = day; DG = dentate gyrus; E = embryonic day; eC = external capsule; EPO = erythropoietin; FB = forebrain; h = hours; Hipp = hippocampus; HTH = hypothalamus; iNO = inhaled nitric oxide; KO IRAK-4 = mice deficient in IL-1 receptor–associated kinase 4 (IRAK-4); NAcc = nucleus accumbens; n.d. = not defined; P = postnatal day; recombinant IL-18BP = Recombinant interleukin–18 binding protein; ROIs = regions of interest (i.e., where cell death is detected); SCWM = subcortical white matter; ST = striatum; STcn = striatal caudate nucleus; TH = thalamus; WM = white matter.*

**TABLE 9:** Cell death in preclinical models of iatrogenic preterm brain injury: caffeine and anesthetics.

**Species**	**Time of the insult**	**Model**	**Cell death markers(time post insult)**	**+ Cell types**	**ROIs**	**Neuroprotectant**	**References**
Rat	E20	**Propofol**, maternal i.v. (8.0 mg/kg) + 1 h (1.2 mg/kg/min)	-c-CASP3 (6 h)	-NeuN	-CTX, TH, HTH	Dexmedetomidine	Li et al., 2016a
	E14	**Sevoflurane,** maternal (3.5%) for 2 h	-↑LC3-II, ↑BECLIN-1, ↓SQSTM1, ↓mTOR, TUNEL, ↓BCL2, (2 h, 12 h, 1 d, 2 d)	-Nestin+	Whole brain	3-MA	Li X. et al., 2017
	P3	**Caffeine**, 1 × i.p. (80 mg/kg)	c-CASP3 (6 h)	n.d.	Whole brain (Hipp, ST, TH, CTX)	–	Cabrera et al., 2017
	P4	**Caffeine**, 1 × /d i.p., for 3 d (10 mg/kg)	TUNEL (6 h, 12 h, 1 d)	n.d.	HTH, DG	–	Endesfelder et al., 2017a
	P6	**Caffeine**, 1 × (10 mg/kg)	PARP-1, AIF (1 d or 2 d)	n.d.	Whole brain	–	Endesfelder et al., 2017b
	P3	**MgSO_4_**, 1 × /h, for 3 h (4 × 250 mg/kg)	c-CASP3, apoptotic by EM	Neurons	CTX, STcn, STp, Hipp, TH, CB c	–	Dribben et al., 2009
	E20	**Propofol**, maternal i.v. (8.0 mg/kg) + 1 h (1.2 mg/kg/min)	-c-CASP3 (6 h)	-NeuN	-CTX, TH, HTH	Dexmedetomidine	Li et al., 2016a
	P4	**Phenobarbital**, 1 × /d i.p., for 3 d (50 mg/kg)	-TUNEL (6 h, 12 h, 1 d)	-n.d.	Frontal CTX, retrosplenial CTX, HTH, TH, DG	–	Endesfelder et al., 2017a

Mouse	E14	**Sevoflurane** (2.5%), 2 h	-c-CASP3	-n.d.	Whole brain	–	(Zheng et al., 2013)
	P6	**Sevoflurane** (3%) or **isoflurane** (2%) or **desflurane** (4/8%), for 2 h/3 h or 6 h	-c-CASP3, c-PARP, TUNEL (18 h) -c-CASP3, TUNEL (2 d)	-n.d	-CX, Hipp, Am, TH, ST, CP -Hipp -Hipp	- -Luteolin -Tanshinone IIA	Kodama et al., 2011 Wang Y. et al., 2019 Xia et al., 2017
	P6	**Sevoflurane** (3%), 1 × /d for 2 h, for 3 d	↑LC3-II, ↓SQSTM1,	n.d	Hipp	3-MA	Wang X. et al., 2019
	P3	-**Midazolam**, 1 × i.p. (6 mg/kg); **ketamine**, 1 × i.p. (40 mg/kg); **fentanyl**, 1 × i.p. (40 μg/kg) **+ caffeine** (80 mg/kg)	c-CASP3 (6 h)	n.d.	Whole brain (Hipp, ST, TH, CTX)	–	Cabrera et al., 2017
	P5–P7	**Propofol**, i.p. (50/100 mg/kg)	c-CASP3	n.d.	STcn, CTX	–	Cattano et al., 2008
Rhesus macaque	100–120 d ga	**Isoflurane** (5 h) 3 h before preterm delivery	c-CASP3	MBP, NeuN	- ST, PT	–	Noguchi et al., 2018
	120 d ga	**Propofol**, i.v. (5 h), 3 h before preterm delivery	c-CASP3 fractin (3 h)	MBP, neurons	CTX, STcn, STp, Am, CB, infCOLL CSO, CC, iC	–	Creeley et al., 2013
	120 d ga 100–120 d ga	-**Isoflurane**, i.v. (0.7–1.5 vol%), 5 h, 3 h before preterm delivery - **+ caffeine** (1 × 80 mg/kg, 1 × /d 20 mg/kg)	-c-CASP3 (3 h) -↑ c-CASP3	-MBP, NeuN -MBP, NeuN	CTX, STcn, STp, Am, CB, -CB, ST	– –	Creeley et al., 2014 Noguchi et al., 2018
	120 d ga	**Ketamine**, i.v. (10 mg/kg) + 5 h (10–85 mg/kg/min)	c-CASP3 (3 h)	n.d.	CTX, STcn, STp, Am, GP, CB	–	Brambrink et al., 2012

*AIF = apoptosis-inducing factor; Am = amygdala, CB = cerebellum; CC = corpus callosum; c-CASP = cleaved caspase; CSO = centrum semiovale, CG = cingulum; CTX = cortex; Cyc = cytochrome c; d = day; DG = dentate gyrus; E = embryonic day; eC = external capsule; EM = electronic microscopy; EPO = erythropoietin; FB = forebrain; GP = globus pallidus; h = hours; Hipp = hippocampus; HTH = hypothalamus; iC = internal capsule, infCOLL = inferior colliculus NAcc = nucleus accumbens; n.d. = not defined; P = postnatal day; ROIs = regions of interest (i.e., where cell death is detected); SCWM = subcortical white matter; ST = striatum; STcn = striatal caudate nucleus; STp = striatal putamen; TH = thalamus; WM = white matter.*

The need to better understand preterm brain damage at the molecular level requires selecting the most appropriate species, age (at which the insult is done), and type of injury, as well as the severity of the insult according to the precise addressed question. Preterm birth is by itself a high-risk factor for brain injury but different deleterious events (alone or combined) can occur and aggravate brain development such as inflammation, hypoxia, ischemia, or hemorrhage. Preclinical models were developed to reproduce one or two of these factors. In the present review, we mainly focused on studies revealing the presence of a type of cell death in the different panels of preterm brain injury models using cell death markers (i.e., studies using more than classical histological observations of pyknosis and karyorrhexis or cell counting).

### Preclinical Models of Periventricular Leucomalacia

Even if, thanks to the important progress in neonatal care, the incidence of cPVL has considerably decreased over the past decades, cPVL remains a major cause of cerebral palsy (Hielkema and Hadders-Algra, 2016; Pierrat et al., 2017). Some models were developed and found to reproduce the formation of cysts (or necrotic foci), i.e., macroscopic areas of massive cell death surrounded by activated microglia suggesting rapid necrotic mechanisms (Debillon et al., 2003).

In a rat inflammatory model, intracerebral injection of LPS formed cysts at the injection site in the corpus callosum with TUNEL+ cells surrounding the necrotic foci (Fan et al., 2013). Models of intrauterine infection in rabbit and sheep have also been shown to induce the formation of WM cysts (25–30% of survivors) accompanied by apoptotic cell death of WM oligodendrocytes (c-CASP3+) (Dean et al., 2009) and, in rabbit, cell death in the CA2 region of the hippocampus (TUNEL+) (Debillon et al., 2003) (Table 3). These models showed not only that a local strong inflammation can lead to cyst formation but also that fetal systemic inflammation can, for some treated animals, induce necrotic foci formation in the WM and/or apoptotic cell death in both oligodendrocytes and neurons. Many *in vitro* studies strongly suggested that activated microglia (M1 phenotype) directly contribute to oligodendrocyte and neuron apoptotic death by releasing reactive oxygen species or TNF-α (Dean et al., 2010; Baburamani et al., 2014).

Beside inflammation, excitotoxicity is one of the most common deleterious mechanisms involved in the formation of cysts. Excitotoxicity consists in a massive intracellular increase of calcium initiated by overactivation of excitatory amino acids receptors (mainly glutamate) (Puyal et al., 2013). In the human preterm brain, the expression of N-methyl-D-aspartate (NMDA) glutamate receptors peaks in pre-oligodendrocytes, allowing the WM to be particularly vulnerable to excitotoxic insult (Jantzie et al., 2015b). Then, the model using intracerebral injection of the glutamate receptor agonist ibotenate (Table 6) has been widely used to reproduce some aspects of human preterm PVL, including WM cyst formation, ventriculomegaly, reduction in brain structure volume, and decrease of WM thickness and myelination (Descloux et al., 2018). Previous studies showed that ibotenate-induced excitotoxicity triggers an important inflammatory response involved in cyst formation (Tahraoui et al., 2001; Dommergues et al., 2003). Concerning cell death type, compiling evidence suggested that apoptosis is importantly involved in this model. One study mentioned the presence of c-CASP3+-positive cells in WM (Neubauer et al., 2016). Neurodegeneration of cortical neurons has also been demonstrated by Fluoro-Jade staining and evidence suggested that caspase-dependent (CASP3) and -independent apoptotic mechanisms (AIF nuclear translocation) were implicated in cortical damage without performing, in most of the studies, an identification of the type of dying cells. Astrocytic cell death has been reported to occur in the site of ibotenate injection (Tahraoui et al., 2001). More recently, we have shown that cortical neurons [(RBFOX3)/NeuN+] presented both activation of CASP3 and nucleus fragmentation (Descloux et al., 2018). Moreover, we have clearly demonstrated that apoptotic neurons displayed mixed morphological features of apoptosis (chromatin condensation) and intense autophagy (increased presence of autophagosomes and autolysosomes). Ibotenate increased both the number of autophagosomes (increased LC3-II and LC3+ dots) and autophagic degradation [p62/SQSTM1 reduction and increased number of autolysosomes (LAMP1- and CATHEPSIN B+ vesicles)]. Inhibition of autophagy with 3-methyladenin (3-MA) prevented CASP3 activation, suggesting a role of autophagy upstream of apoptosis as previously shown in other models (Puyal et al., 2009; Grishchuk et al., 2011; Xie et al., 2016). Interestingly, 3-MA afforded strong protection by reducing not only cortical lesion but also WM deficits, indicating that preventing primary neuronal death by autophagy inhibition could reduce WM damage (Descloux et al., 2018).

Lesions mimicking human PVL were also observed in a sheep model of severe hypoxia–ischemia (Table 4) involving a double hit protocol and producing WM necrotic foci related to GM (CX and TH) damage (transient maternal hypoxia and brachiocephalic artery occlusion followed by a second hypoxia 6 h later) (Hagen et al., 2014).

### Preclinical Models of Diffuse White Matter Injury

While hypoxia/ischemia represents one of the major risk factors for neonatal encephalopathy in term babies, for preterm, however, inflammation has been of, in the last decade, growing importance in the field. The role of a hypoxic event in preterm brain injury has, indeed, no clear evidence. Argument that is more rigorous is needed today before claiming hypoxic–ischemic encephalopathy such as measurement of hypoxemia and metabolic acidosis (Graham et al., 2004; Gilles et al., 2017; Paneth, 2018). It is now proposed that inflammation could be a leading cause of preterm brain damage (Gilles et al., 2017). In human preterm babies, chorioamnionitis and especially postnatal sepsis are associated with WM injury and adverse neurodevelopmental outcomes (Shah et al., 2008; Anblagan et al., 2016; Bierstone et al., 2018; Heo et al., 2018). Moreover, inflammation is a common process in different preterm brain injuries either directly as in intrauterine infection/chorioamnionitis or secondarily because of a primary insult such as hemorrhage or hypoxia/ischemia. Despite the current questioning of the role of hypoxia in preterm brain damage, most of the studies investigating cell death mechanisms in diffuse WM injury have been performed in models involving both stimuli: inflammation (Table 3) and hypoxia/ischemia (HI) (Tables 4, 5) (Jantzie and Robinson, 2015).

#### Preclinical Inflammatory Models

Direct inflammatory lesions are induced in most of the preclinical models by an acute or chronic exposure to either gram-negative bacteria lipopolysaccharide (LPS) endotoxin or more recently by injection of interleukin-1β that leads to a persistent inflammatory response, astrogliosis, and myelination deficits (Dean et al., 2015). It has been shown that exposure to inflammation during the perinatal period could alter transiently or permanently neurodevelopmental outcome (learning and motor difficulties and development of psychiatric disease such as schizophrenia or autism) (Fan et al., 2005b; Boksa, 2010; Rousset et al., 2013; Schaafsma et al., 2017; Straley et al., 2017; Zhang et al., 2018). Cell death has been mostly investigated in models of fetal infection in rat, sheep, and macaques in which apoptotic cell death (c-CASP3) was found to be scattered, located essentially in WM, and observed mainly after days following inflammatory insult (Table 3). When investigated, cell type identification indicated that CASP3 activation occurred in oligodendrocyte lineage (Olig2, CNPase, and PDGFαR). However, injury of the GM was also described. A study in sheep involving intra-amniotic LPS injection also described CASP3 activation in the cortex and the hippocampus 8 days after LPS exposure (Gussenhoven et al., 2018). Another study involving i.p. LPS injections in rat dam (at E18 and E19) showed the presence of TUNEL and c-CASP3-positive cells in the periventricular striatum of pups 7 days after birth (Rousset et al., 2006).

An intracerebral injection of LPS consists of much more severe inflammatory models by mimicking late conditions of pathogen infiltration after the breakdown of the blood–brain barrier. In a model of LPS exposure in postnatal rat (P3), the production of the CASP-dependent fragment of β-actin, fractin, was observed in the subcortical WM (SCWM) as well as in axonal fascicles of striatum (Lodygensky et al., 2014; Ginet et al., 2016), suggesting a LPS-induced axonopathy (Sokolowski et al., 2014). The mechanisms involved in apoptosis induction could depend on the cell type. In fact, in a model of intracerebral injection of IL-1β in P5 rats inducing a global CASP3 activation, TUNEL staining was observed in Lectin+ microglia, in O4+ and O1+ oligodendrocyte precursors, and in some NF+ neurons 1 day after the insult (Cai et al., 2004). Inflammation-related cell death was partially associated with excitotoxicity since the AMPA/kainate receptors antagonist NBQX reduced the global number of TUNEL+ cells without protecting oligodendrocyte precursors. This could be explained by an age-dependent sensitivity of WM pre-oligodendrocytes to AMPA toxicity that occurs later at P7 (Follett et al., 2000).

A single study investigated autophagy in an inflammatory model (Carloni et al., 2016), showing that, when LPS was injected in the rat dam (at E18 and E19), autophagy appeared impaired (LC3-II and p62/SQSTM1 levels were increased in P1 brain rat pups). However, this study evaluated the overall autophagy response on whole brain extracts, a method that limits the conclusions concerning the effect on autophagy according to either WM, GM, or the cell type.

Very recently, an involvement of necroptosis has been proposed to occur after LPS-induced brain inflammation in P3 old rats by showing an increase in necroptotic gene expressions (RIPK1, RIPK3, and MLKL) 24 h after the insult (Pierre et al., 2019).

#### Hypoxic–Ischemic Models

Even if the role of HI has become a subject of debate (Gilles et al., 2017), it remains one of the most used models for studying preterm brain injury that induces both severe WM and GM injury and are then used mainly as a model of cPVL. Cell death has been investigated mainly in sheep and rodent (Table 4).

Sheep models consist in inducing a hypoxic–ischemic event in the fetus by either umbilical cord occlusion or by brachiocephalic arteries occlusion combined with maternal hypoxia. CASP3 activation, pyknotic nuclei, as well as TUNEL staining were described mainly in PVWM (periventricular and subcortical WM) and SCWM but also in cortex, basal ganglia (caudate nucleus and putamen), and less frequently in thalamus, hypothalamus, and hippocampus. One study investigating cell death within the first 3 h following HI showed that necrotic features are detected early, from time zero onwards, without signs of apoptosis in different cerebral regions (mesencephalon, cerebellum, pons, basal ganglia, cortex, hippocampus, thalamus, and hypothalamus) (Goni-de-Cerio et al., 2007). From 3 h onwards, apoptotic dying cells are detected and not restricted to cerebral regions where necrotic neurons were histologically observed at 0 h (mesencephalon, cerebellum, pons, and basal ganglia). Interestingly, some early necrotic regions will then become strongly apoptotic later (especially cortex, cerebellum, and pons), suggesting that different cell death processes are involved sequentially in the same damaged cerebral region. However, since all these observations are focused on unregulated necrosis, it could be speculated that regions displaying either apoptotic or necrotic features at later time points are regions where delayed cell death is prominent, including potentially not only apoptosis but also a regulated form of necrosis such as necroptosis.

The subplate zone is a transient layer of the developing cerebral cortex that plays a highly important role in the structural brain development and its plasticity. It contains different neuronal cells and especially early-generated subplate neurons whose cell bodies are located in developing WM. Subplate injury has been since years discussed in EoP and is becoming more and more important over the last decade. When sheep maternal hypoxia and fetal ischemia were induced early (92d GA), CASP3 activation was also present in the subplate but essentially in oligodendrocytes, suggesting a more important resistance of subplate neurons in sheep (McClendon et al., 2017). On the contrary, selective vulnerability of subplate neurons was described in the most widely used model of HI in P2–P3 rodents (uniCCAO followed by systemic hypoxia) (McQuillen et al., 2003; Mikhailova et al., 2017). However, another study could not confirm a specific vulnerability of subplate neurons compared to other deep layers or to the WM (Okusa et al., 2014). It is important to point out that these studies have evaluated only the density of neurons in the subplate and did not perform co-labeling with cell death markers, which is not sufficient to conclude for a specific neuron death in the subplate since an impairment in neuronal differentiation could also be involved. In a different rat model consisting in placental transient hypoxia–ischemia at E18 (uterine arteries occlusion), however, the presence of TUNEL+ neurons has been reported in the subplate at P2 (Jantzie et al., 2015a).

Even if widespread CASP3 activation or TUNEL labeling were shown in models of HI in P1 to P6 rodents in both GM (cortex, thalamus) and WM (Segovia et al., 2008; Jablonska et al., 2012; Scafidi et al., 2014; Seyama et al., 2018), few studies characterized the type of dying cells. In a mouse model of chronic hypoxia (8 days at 10.5% O_2_ from P3), oligodendrocytes were shown to undergo CASP3-dependent apoptosis even 1 week after the end of hypoxic treatment (Jablonska et al., 2012). In the model of HI in P3 rats pups, Segovia and colleagues (Segovia et al., 2008) showed that, in addition to pre-oligodendrocyte maturation arrest, O4+ cells underwent mainly caspase-independent cell death mechanisms (c-CASP3 negative but morphological characteristics of degeneration) 24 h after the insult, whereas 4 days later, they died mostly through caspase-dependent pathways, suggesting that different forms of cell death are involved in acute or delayed cell death of oligodendrocytes (Segovia et al., 2008).

In a model of anoxia in P1 rat pups, damaged hippocampus showed cell death with EM mixed morphological features of apoptosis, necrosis, and enhanced autophagy (Takada et al., 2015), suggesting that more than one cell death type could be involved in hypoxic–ischemic brain damage with a continuum of cell death that could include the three cell death types as it has been proposed in models of HI in more mature P7 brain (Portera-Cailliau et al., 1997; Ginet et al., 2009a; Northington et al., 2011). Interestingly, this study showed that whereas CA2/CA3 presented a neurodegeneration revealed by both Fluoro-Jade B+ staining and EM ultrastructure study, this hippocampal region was TUNEL negative and presented no more CASP3 activation than in control. This result points out the necessity to study cell death with alternative markers of cell death, different from CASP3 activation (that is moreover developmentally expressed in the immature brain) and TUNEL staining.

In summary, in inflammatory and/or HI premature models, cell death has been investigated in different models and species, and is present in cortical and deep GM neurons and in different glial cells (OL and microglia) with different cell death features reported (necrosis, apoptosis, and autophagic cell death).

### Preclinical Models of Intraventricular and Intraparenchymatous Hemorrhages

Very few studies have investigated cell death occurring after IVH in preclinical preterm models (Table 7). Cell death has been studied in two main models reproducing the conditions of germinal matrix hemorrhage (GMH) (germinal matrix ruptures through the ependyma into the lateral ventricle) leading to IVH in preterm human brains. The first approach consists of applying periventricular or intracerebroventricular injections of blood (from dam or autologous) in rodent pups (P1 to P4). The second approach is to perform an i.p. injection of glycerol in rabbit fetus (29 days GA) that produces an intracranial pressure through profound osmotic changes followed by a reperfusion leading to GMH that can extend to the lateral ventricle (Table 7). In both models, pups developed severe IVH associated with progressive post-hemorrhagic hydrocephalus (PHH) compatible with grade 3 or grade 4 IVH in humans, even if the percentage of induced PHH is variable. The IVH pups displayed impaired sensorimotor functions, inflammation, defects in myelination, and increased levels of inflammatory cytokines in the CSF.

Increased cell death and apoptosis have been evidenced by TUNEL staining and CASP3 activation in the periventricular WM (Dummula et al., 2011; Ahn et al., 2013; Gram et al., 2014; Dohare et al., 2016; Mukai et al., 2017) and adjacent GM [striatum (Xue et al., 2003) or cortex (Georgiadis et al., 2008; Zia et al., 2009; Vinukonda et al., 2010)]. However, the identification of the dying cells has not been determined except in some studies showing that cell death (Fluoro-Jade+ cells) could affect O4+ pre-oligodendrocytes or neurons.

### Preclinical Models of Iatrogenic Preterm Brain Injury

#### Hyperoxia

The involvement of cell death and apoptosis in preterm brain injury is well documented in preterm models of hyperoxia in postnatal rodent (from P0 to P6) (Table 8). Hyperoxia was shown in those models to affect both WM and GM (cortex, striatum, thalamus, and hippocampus) by promoting cell death of oligodendrocytes (Gerstner et al., 2006, 2007; Schmitz et al., 2014; Hoeber et al., 2016) and neurons (Yis et al., 2008a, b; Endesfelder et al., 2014). The involvement of apoptosis as a major cell death process was demonstrated by the implication of not only CASP3 but also numerous molecular markers of both intrinsic (Cyc, AIF, and APAF-1) and extrinsic (Fas, FasL, FADD, CASP8, and BID) apoptotic pathways inducing caspase-dependent (CASP1, 2, 3, and 8) and independent (AIF) mechanisms.

Furthermore, Bendix and colleagues (Bendix et al., 2012) showed an effect of hyperoxia on autophagy during the first 24 h of hyperoxia in the developing rat preterm brain. Some important autophagy-related genes (atg) for autophagosome formation were upregulated (BECLIN1, ATG3, ATG5, ATG12, and LC3-II) (Bendix et al., 2012) at 12 h after hyperoxia treatment. However, we cannot conclude on the effect of hyperoxia on autophagic flux (active autophagic process of degradation) since efficiency of the degradative part of autophagy was not investigated. Increased autophagosome presence could result from enhanced autophagic flux as well as impaired lysosomal degradation (Puyal et al., 2013; Klionsky et al., 2016). While this study showed that autophagy is certainly affected by hyperoxia, more investigations (assessing the state of the flux and its function) are needed before to conclude on the role of autophagy in hyperoxia-mediated cell death.

#### Caffeine

It has been shown that caffeine treatment (10 to 80 mg/kg, i.p.) activated apoptosis (CASP3, AIF) and increased TUNEL staining in the brain of rat pups (P3, P6) (Table 9) (Cabrera et al., 2017; Endesfelder et al., 2017a, b). Recently, Noguchi and colleagues showed that caffeine treatment in fetal macaque (110–120 days GA) anesthetized with isoflurane strongly sensitized brain (CX, ST, and CB) against isoflurane-induced apoptosis, especially NeuN-positive neurons, more than MBP-positive oligodendrocytes (Noguchi et al., 2018).

#### Anesthetics

Different studies demonstrated pro-apoptotic effects of anesthetics [especially propofol, ketamine, and ethers (isoflurane, sevoflurane)] on immature brain of rodents and macaques (Table 9). Combination of anesthetic with caffeine is a powerful aggravating factor (Noguchi et al., 2018). These anesthetics have been shown to trigger widespread CASP3 activation or to increase TUNEL labeling in neurons and oligodendrocytes (Cattano et al., 2008; Dribben et al., 2009; Brambrink et al., 2010, 2012; Creeley et al., 2013; Li et al., 2016a; Xiong et al., 2016) associated with some cognitive deficits. In two studies using sevoflurane, autophagy was shown to be enhanced, in addition to apoptosis, in immature brains, and autophagy inhibition using 3-MA was both anti-apoptotic and neuroprotective (Li X. et al., 2017; Wang X. et al., 2019).

## Conclusion and Future Prospects

In conclusion, common and converging observations from animal models and human brains from autopsy suggested that cell death mechanisms in preterm brain injuries mainly involve apoptotic mechanisms. Although most of the studies missed to identify the type of cells subjected to cell death, some suggested that different cell types are affected depending not only on the severity and the type of the insult but also on the time point investigated. In mild preterm injuries, the cell death appeared to be diffuse and restricted (when detected) to preOL, whereas in more severe cases, cell death could affect almost all cell types including neurons.

Nevertheless, it appears that, although cell death is an ineluctable event occurring in severe preterm brain injuries such as cPVL and IVH, but not necessarily in dWMI, cell death has not been deeply investigated and characterized either in preclinical models or in tissues from human preterm neonates. The importance of apoptosis versus other cell death pathways (such as regulated necrotic and autophagic) is certainly overestimated since almost all the studies used only apoptotic (such as c-CASP3 and TUNEL) markers as indicators of cell death. Moreover, by using only c-CASP3 and TUNEL markers, cell death occurrence can be simply underestimated or wrongly declared absent since cell death types independent of CASP3 and not inducing (at least in an early stage) DNA fragmentation exist. In the model of anoxia in P1 rat pups, damaged hippocampal CA2/CA3 showed, for example, cell death mechanisms mainly independent of CASP3, not positive for TUNEL and with EM morphological features of necrosis and enhanced autophagy (Takada et al., 2015). We have previously clearly demonstrated the presence and the prodeath role of enhanced neuronal autophagy in models of both term (Ginet et al., 2009b; Puyal et al., 2009; Ginet et al., 2014a; Xie et al., 2016) and more recently preterm (Descloux et al., 2018) brain injury. We showed that, after neonatal cerebral HI, rat hippocampal dying CA3 neurons displayed high autophagic features without apoptosis activation, i.e., TUNEL and CASP3 negative (Ginet et al., 2009b). In addition, the discovery of programmed forms of necrosis and some of its molecular actors will allow one to investigate the implication of regulated necrosis. Necroptosis is especially concerned since this type of cell death is known to be activated by inflammatory signals.

Since “we find what we are looking for”, one could speculate that most of the studies missed to identify cell death mechanisms involved in the pathogenesis of the studied preterm brain damage. The increasing advances made to understand the biochemical mechanisms of the different cell death pathways and the recognition of regulated necrosis and autophagic cell death lead to switch, over the last 10 years, in the identification of the cell death type from a purely morphological to a molecular analysis (Galluzzi et al., 2012). Today, many markers of the different types of cell death could be used in human samples to systematically evaluate the presence not only of apoptosis but also of other forms of cell death including regulated necrosis and autophagic or autophagy-mediated cell death. Expression and activation of RIPK3 and MLKL could be investigated as markers for necroptosis. Concerning autophagic or autophagy-mediated cell death, an increase in autophagosome presence alone is not enough to demonstrate an increased autophagic flux since it could result from a lysosomal degradation failure. Then, an increase in punctate LC3 immunolabeling (marker of autophagosomes) together with an increase lysosomal activity, evaluated by either a decrease in p62/SQSTM1 labeling (selectively degraded by autophagy) or an increase in the size and number of vesicles labeled by lysosomal markers such as LAMP1 or different cathepsins (presumably autolysosomes), should be used to detect enhanced autophagy. Moreover, since increased autophagy could be either a prosurvival or a prodeath mechanism, conclusions on the role of enhanced autophagy in cell death has to be taken with caution due to the duality of autophagy in cell survival (protective vs. destructive) depending the conditions and cell type.

Future studies designed to provide a more precise characterization of the molecular cell death mechanisms and also identification of dying cell types will allow a better understanding of the preterm brain pathogenesis and will certainly participate to develop new and efficient protective strategies to prevent or treat injuries of the preterm human brain.

## Author Contributions

AT, VG, and JP conceived, wrote the manuscript, drew the tables and figures, and critically revised the manuscript.

## Conflict of Interest

The authors declare that the research was conducted in the absence of any commercial or financial relationships that could be construed as a potential conflict of interest.

## Abbreviations

3-MA: 3-methyladenin
ADC: apparent diffusion coefficients
ATG: autophagy-related gene
CASP: caspase
c-CASP: cleaved caspase
cPVL: cystic periventricular leucomalacia
CSF: cerebrospinal fluid
dWMI: diffuse white matter injury
EM: electronic microscopy
EoP: encephalopathy of prematurity
ER: endoplasmic reticulum
FA: fractional anisotropy
GA: gestational age
GM: gray matter reduction
GMH: germinal matrix hemorrhage
HI: hypoxia–ischemia
IVH: intraventricular hemorrhage
LC3: microtubule-associated protein 1 light chain 3
LPS: lipopolysaccharide
MRI: magnetic resonance imaging
OL: oligodendrocytes
PE: phosphatidylethanolamine
PHH: post-hemorrhagic hydrocephalus
preOL: pre-oligodendrocyte
PVL: periventricular leucomalacia
PWM: periventricular white matter
SCWM: subcortical WM
SVZ: subventricular zone
VPT: very preterm infants
WM: white matter
WMI: white matter injury.
